## [Transparent Peer Review file · Nature Communications]

External Acidity as Performance Descriptor in Polyolefin Cracking using Zeolite-Based Materials

Corresponding Author: Professor Bert Weckhuysen

Version 0:

Reviewer comments:

Reviewer #1

(Remarks to the Author)

In this manuscript, the authors designed various zeolite materials to investigate their differences in catalyzing polymers and small molecules, providing valuable insights into the structure-activity relationship for polymer catalytic cracking and proposing a design for efficient catalysts. However, several issues need to be adequately addressed before the manuscript can be accepted:

Fig. 1 a-b: The authors observed that ZY55 and ZY14 materials exhibited different activities in DMP and the lowest cracking temperatures in PP degradation. An explanation or hypothesis is needed here, rather than simply stating that the catalytic effects of the two materials do not follow a pattern. The manuscript would benefit from a deeper discussion of the underlying mechanisms.

Fig. 1d: The external acid concentration of the ZY35st catalyst is shown to be higher than the overall acid concentration, which seems counterintuitive. Additionally, the figure indicates that the external BAS concentration of most ZY catalysts is very similar, suggesting that PP cracking activity is related to external acid concentration. However, catalysts like ZY14 and ZY5, which have external acid concentrations similar to those of gas-phase zeolites, exhibit significantly lower activity. This discrepancy requires a more detailed and reasonable explanation.

Assessment of Activity: In the fourth paragraph of this section, the manuscript mentions that the polymer faces transport limitations and can hardly enter the pores. The evidence provided for this assumption is weak. Stronger data or calculations are necessary to support this hypothesis and to substantiate the claim.

Fig. 2d-e: The manuscript discusses the difference in E_a for DMP and PP. It would be helpful to determine whether this perspective can be derived through calculations using the Benson group theory. Including calculations for both polymers and small molecules could further substantiate the observed differences in E_a and enhance the credibility of the findings.

Isothermal Cracking of Polypropylene: In this section, the manuscript suggests that catalytic activity is related to factors such as loading and neglects the impact of viscosity on the polymer. However, viscosity and entropy should be significant factors in this system, especially since, for small molecules, higher temperatures almost entirely convert them into gaseous products, where viscosity and entropy differ significantly from those of polymers. A more thorough consideration of these factors is necessary.

Polymer Reaction Models: In the second paragraph of the "Isothermal Cracking of Polypropylene" section, two polymer reaction models are mentioned: one where the polymer enters the zeolite pores and undergoes end-group cracking first, and the other where the polymer preferentially cracks on the external surface. These scenarios can be detected by monitoring the spatiotemporal evolution of the products using GC-MS. Complete gas data should be provided in the supplementary information to reflect these observations and support the proposed models.

Model for Designing Acid Sites: In the third paragraph of the "Isothermal Cracking of Polypropylene" section, a model for designing acid sites is mentioned, assuming an ideal hexagonal structure. However, SEM images show that the material used by the authors differs from this ideal hexagonal structure, which could significantly impact the final calculation results. It is recommended that the authors either modify the formula to reflect the actual material structure or synthesize more

uniformly structured materials. Additionally, regarding the configuration in which PP participates in the cracking reaction, the manuscript would benefit from including any relevant literature or computational models that indicate its configuration on the catalyst surface. This information should be included in the supplementary materials.

Reviewer #2

(Remarks to the Author)

This paper describes the application of polyolefin cracking which is a topic of considerable importance. The results are impressive that the polyolefin cracking reaction is initiated outside of the micropores of the catalyst, and mediated largely by hydride transfer, as opposed to direct alkane activation by the acid sites. Prior to publication some important parts of the work need to be clarified.

1. In Fig. 1b, it is reported that the lower the zeolite BAS density, the weaker the catalytic cracking ability. Please explain why ZY55 shows the highest cracking activity during pp cracking?
2. In page 3, the authors state that the external acidity of the catalyst is more favorable for polyolefin conversion, can it be further demonstrated experimentally that the internal acid sites of the zeolite have less of an effect?
3. Are the TGA calculation predictions subject to large errors because of the large differences between the TGA ramp experiments and the semi-intermittent reactor experimental processes (including warming rates, reaction conditions, etc.)?
4. In Fig. 3e, SEM cannot clearly show that the extended polymer chains can cover a large portion of the zeolite particles, please provide additional characterization to prove.
5. In Fig. 4b, explain why C4 and C5 have the highest selectivity in the gas?

Version 1:

Reviewer comments:

Reviewer #1

(Remarks to the Author)

The authors addressed all my concerns. I strongly recommend the paper for publication.

Reviewer #2

(Remarks to the Author)

In this contribution, the authors report on the structure-composition-property relationship between zeolite-based materials and polyolefin cracking. The research area is of high relevance, being timely and a hot topic at the moment. In general, the paper is very solid: the authors demonstrate plastic cracking activity using ultrastable zeolite Y materials does not depend on the bulk Brønsted acid site content, but rather on the concentration of acid sites located on the outer surface and in mesopores. In my opinion, this paper could be published in Nature Communications.

Point-by-point response to the reviewers' comments

Comments are labeled in blue. Changes to the manuscript are marked green.

Reviewer 1

Fig. 1a-b: The authors observed that ZY55 and ZY14 materials exhibited different activities in DMP and the lowest cracking temperatures in PP degradation. An explanation or hypothesis is needed here, rather than simply stating that the catalytic effects of the two materials do not follow a pattern. The manuscript would benefit from a deeper discussion of the underlying mechanisms.

The discrepancy in activities between these different catalyst materials is indeed one of the core findings of this work. This led us to the hypothesis stated in line 4-5 of the first paragraph of page 3:

“...we hypothesized that polymers enter the micropores of ZY very inefficiently or not at all. PP cracking activity might therefore be more dependent on the number of acid sites located outside of micropores, that is in mesopores and the external surface of zeolite crystals.”

This hypothesis is then supported by determination of the non-micropore acid site density determined by TTBP-IR (Fig. 1c,d).

Further explanations for differences in activity are given in the section “Rationalizing the varying effect of catalyst loading” (starting page 6). In these sections we propose that two additional explanations for differences in plastic cracking activity among the different zeolite materials, namely different distributions of external acid sites as well as pre-cracking on external sites followed by further cracking in micropores.

The main manuscript was adjusted to make this clearer:

Page 3

This result implies that activity trends obtained by cracking of shorter model hydrocarbons like hexane or DMP have limited predictive power for polyolefins cracking and is evidence of deviating structure-composition-performance relationships between the two chemical reactions, which will be elucidated in this work.

Page 4

This explains the similarity in the observed PP cracking activities (Fig. 1b), and demonstrates that external acidity is a more appropriate descriptor for polyolefins conversion activity, although not without restrictions, as e.g. ZY₁₄ and ZY₅₅ show similar external acidities but different activities at low loading (*vide infra*).

Regarding mechanistic differences, there is no reason to assume the molecular elemental steps of hydrocarbon cracking differ between smaller and larger hydrocarbons. However, the relative prevalence of mono vs. bimolecular cracking could indeed be different, which we discuss in the section “isothermal cracking of polypropylene”.

“Therefore, the polyolefins cracking reaction could be mainly propagated by hydride transfer, which has been shown to express a lower energy of activation compared to monomolecular cracking.”

Fig. 1d: The external acid concentration of the ZY35st catalyst is shown to be higher than the overall acid concentration, which seems counterintuitive.

In Figure 1d, bulk and external acidities appear similar but are indeed very different. This is clear when one realizes the bulk and external acidities refer to different y-axes (with the right one referring

to the external acidity and the left describing the bulk acidity, as indicated by the color of the labels). This was done to be able to show the strong similarity and small deviations in different external acid site densities among the zeolite materials, which are not as evident when the axes would be scaled identically.

We have adjusted the figure to make the varying axes clearer by adjusting the color of the tick labels.

Page 3

Additionally, the figure indicates that the external BAS concentration of most ZY catalysts is very similar, suggesting that PP cracking activity is related to external acid concentration. However, catalysts like ZY14 and ZY5, which have external acid concentrations similar to those of gas-phase zeolites, exhibit significantly lower activity. This discrepancy requires a more detailed and reasonable explanation.

The external BAS concentration can indeed not explain all differences in activities observed, as mentioned for catalysts ZY55 and ZY14 on page 4 line 10. The extensive experiments and simulations described in the sections ‘isothermal cracking of polypropylene’ and ‘Rationalizing the varying effect of catalyst loading’ attempt at explaining precisely these deviations.

However, we did not comment on ZY14 and ZY5 specifically, where a critical difference is the lack of steam treatment for ZY5. Steaming can drastically increase the cracking activity of a Y-zeolite, which appears also to be the case for plastic cracking. We have adjusted the main manuscript to also discuss this point.

Page 4

Importantly, the external acidity fails to capture the remaining activity trends. ZY55 and ZY14 showed similar external acidities, yet their T_{max} at low catalyst loading deviated by >20 °C (Fig. 1b). Similarly, the non-steamed ZY5 shows comparable external acidity but noticeably lower activity compared with the other zeolites. However, here the decreased activity might be explained in a similar fashion as its reduced gas cracking activity (Fig. 1a), namely by the lower amount of mesoporosity of the non-steamed ZY5 compared to the other zeolites and the higher acid strength of steamed zeolites.

Assessment of Activity: In the fourth paragraph of this section, the manuscript mentions that the polymer faces transport limitations and can hardly enter the pores. The evidence provided for this assumption is weak. Stronger data or calculations are necessary to support this hypothesis and to substantiate the claim.

To what extent polypropylene macromolecules of a given molecular weight and branching structure can enter into micropores is indeed not a settled question, which is why the statement mentioned was explicitly stated as a hypothesis justifying further experiments (i.e. not an assumption). From page 4:

*“We recently demonstrated that plastic cracking is subject to multiple types of transport limitations,²³ and together with findings from earlier literature,¹⁴ we **hypothesized** that polymers enter the micropores of ZY very inefficiently or not at all.”*

In our opinion there is ample evidence for highly restricted movement of macromolecules in micropores:

- 1. Transport limitations are already observed for fluid catalytic cracking catalysts with a mesoporous shell¹ and it is reasonable to expect that transport in micropores of a zeolite will be even more restricted.*
- 2. The diffusion coefficient of smaller hydrocarbons in silicalite decreases exponentially with growing carbon number.²*
- 3. The viscosity of a confined polymers can be more than 2 order of magnitude larger compared to bulk if the pore radius exceeds the radius of gyration of the polymer,³ which could inhibit transport further.*
- 4. If the polymer enters into a micropore channel, side chains caused by branching could prohibit further entry into the pore network, in analogy to pore mouth catalysis.⁴*
- 5. When PP is adsorbed on a steamed US-Y zeolite (CBV780, ZY₅₅ in this study), the capacity of the zeolite to adsorb PP corresponds to only 5% of the pore volume of the catalyst.⁵*

We have amended the main manuscript to include these prior findings and added the respective references.

Page 3

We recently demonstrated that plastic cracking is subject to multiple types of transport limitations,²³ and ~~together with findings from earlier literature,¹⁴~~—we hypothesized that polymers enter the micropores of ZY very inefficiently or not at all. This hypothesis is supported by multiple key findings in the literature. Most critically, Macko and coworkers determined the capacity of a CBV780 Zeolite (ZY₅₅ in this study) in the adsorption of PP from solution in units of gram of polymer per gram of zeolite.³¹ Combining their capacity with the pore volume of our sample and the bulk density of polypropylene, we estimate that only around 5% of the total pore volume or 10% of the micropore volume would be occupied by polymer. We note that adsorption from solution will most likely be easier than from the melt, as the polymer chains in solution are not as entangled as in the melt. Therefore, polymer most likely only ‘plugs’ micropore entries, without properly entering the pore system. Furthermore, side chains resulting from branching might also sterically prohibit the polymer from entering beyond a certain length, in analogy to pore mouth catalysis.³² Drawing on evidence from gas phase cracking, the diffusion coefficient of linear hydrocarbons in a silicalite molecular sieve decreases exponentially with increasing carbon number,³³ suggesting that diffusion will be severely limited for polymers. Furthermore, it was demonstrated that the viscosities of polymers can increase up to two orders of magnitude under confinement.³⁴

Fig. 2d-e: The manuscript discusses the difference in E_a for DMP and PP. It would be helpful to determine whether this perspective can be derived through calculations using the Benson group theory. Including calculations for both polymers and small molecules could further substantiate the observed differences in E_a and enhance the credibility of the findings.

Benson group increment theory allows to estimate enthalpies of formation of molecules based on their constituting functional groups, and therefore make thermodynamic predictions. The activation energy however is a kinetic parameter. To our best knowledge activation energies can not be derived from this approach.

Polymer Reaction Models: In the second paragraph of the "Isothermal Cracking of Polypropylene" section, two polymer reaction models are mentioned: one where the polymer enters the zeolite pores and undergoes end-group cracking first, and the other where the polymer preferentially cracks on the external surface. These scenarios can be detected by monitoring the spatiotemporal evolution of the products using GC-MS. Complete gas data should be provided in the supplementary information to reflect these observations and support the proposed models.

The approach suggested could in theory be indeed the strongest evidence for this hypothesis, and we have considered it as well. Unfortunately, primary, i.e., larger cracking products can react further in micropores of the zeolite, shifting the selectivity. When comparing gas yields as well as size distributions of the alkanes and alkenes in the resulting pyrolysis oils, the catalysts with higher gas cracking activity like ZY₁₂ show higher gas yields and overall shorter hydrocarbons in the pyrolysis oil, indicating that for this set of catalysts the selectivity observed is strongly influenced by the bulk acidity. In the 'conclusions' section we outline goals for future studies that could help understand the underlying mechanism better.

We included comments with regards to selectivity analysis in the main manuscript and added a figure showing cumulatively integrated masked 2D-chromatograms to the SI.

Page 7

If active sites are accumulated together, more chain end scission could be expected, while a more homogenous distribution could cause more evenly distributed cracking probability along the polymer backbone (Fig. 3c). Potential testing of this hypothesis by selectivity analysis is discussed in the following section.

Page 8

This implies that tuning of the reaction selectivity towards products like aromatics cannot be achieved by simply adjusting the bulk acidity of ZY in a semi-batch reactor. The size distribution of the resulting products might provide evidence for preferential cleavage of different bonds along the polymer backbone as discussed in the previous section. For more chain-end scission, one would expect overall lighter products. However, the formed product vapors can still react with the surrounding zeolite, including its micropores, and would therefore be cracked further. In this case, the product size distribution would be noticeably affected by the gas-cracking ability of the given catalyst. As mentioned, more light gases are observed for the catalyst with the highest gas cracking activity, and when qualitatively comparing boiling point distributions of the alkane and alkene products in the pyrolysis oil by masked and integrated 2D-chromatograms (Fig. S13), it can also be seen that for catalysts with a higher gas-cracking activity the selectivity shifts towards lighter hydrocarbons. This consecutive cracking prohibits testing whether preferential cleavage of certain bonds is taking place by means of selectivity analysis in this reactor configuration. We expect that reaction temperature and residence time in the reactor will play a more important role in determining the selectivity pattern.

Fig. S13: Comparison of boiling point distribution of alkanes/alkenes in the pyrolysis oils by integration of masked 2D chromatograms.

Model for Designing Acid Sites: In the third paragraph of the "Isothermal Cracking of Polypropylene" section, a model for designing acid sites is mentioned, assuming an ideal hexagonal structure. However, SEM images show that the material used by the authors differs from this ideal hexagonal structure, which could significantly impact the final calculation results. It is recommended that the authors either modify the formula to reflect the actual material structure or synthesize more uniformly structured materials.

We assume the reviewer is referring to supplementary note 4. Herein, we try to make the argument that a polymer chain can interact with multiple acid sites simultaneously. For this we attempt to determine the distance between external sites for a given external acid site density and external surface area. An upper bound for the nearest-neighbor distance is reached when the acid sites are spaced equally, e.g., on a hexagonal grid. A comparison between this estimated nearest neighbor distance and the chain length of the polymer supports our claim. In fact, in any other configuration of acid sites the nearest neighbor distance will be shorter. We believe that this argument is sufficient to show that it is not unreasonable to expect a polymer chain to interact with multiple acid sites simultaneously.

Additionally, regarding the configuration in which PP participates in the cracking reaction, the manuscript would benefit from including any relevant literature or computational models that indicate its configuration on the catalyst surface. This information should be included in the supplementary materials.

To our best knowledge, no studies relying on computational methods capable of modeling melt behavior (e.g. molecular dynamics) have been conducted on the configuration of polymer chains located at the melt-zeolite interface. Furthermore, it has been shown⁶ that to describe hydrocarbon zeolite interaction accurately, very high levels of theory are required. As we are unsure which precise question raised in this work could be answered by such a study, we believe this type of simulation to be out of scope for this work.

Regarding experimental studies, PP adsorption on zeolites from solution has been studied, which pointed to a very strong interaction with the zeolite surface and limited penetration into micropores, as referred to above.⁵

Reviewer 2

In Fig. 1b, it is reported that the lower the zeolite BAS density, the weaker the catalytic cracking ability. Please explain why ZY55 shows the highest cracking activity during pp cracking?

The bulk BAS density is critical for cracking of light hydrocarbons (Fig. 1a). The core finding of this work is that for cracking of bulky hydrocarbons like plastics, the external, i.e., non micropore acid site density is a more appropriate descriptor, which is discussed at length in the results section. ZY55 specifically shows the highest PP cracking activity due to its high external acidity (Fig. 1d), however the external acidity cannot capture all kinetic differences upon changes in catalyst loading. Potential explanations for this observation are discussed in the section “Rationalizing the varying effect of catalyst loading”.

Page 4:

*“While the bulk acidity consistently decreases with increasing SAR, the external acidity is remarkably similar for all untreated catalyst materials. This explains the similarity in the observed PP cracking activities (Fig. 1b), and demonstrates that external acidity is a more appropriate descriptor for polyolefins conversion activity, although not without restrictions, as e.g. ZY₁₄ and ZY₅₅ show similar external acidities but different activities at low loading (*vide infra*). To show how this result can be used to synthesize more active cracking catalysts, we aimed at introducing more external acid sites on a given catalyst. ZY₁₄ was subjected to mild steaming (500 °C, 2 h, followed by HNO₃ leaching) yielding the ZY_{35st} sample. The steaming treatment reduces the DMP cracking activity by reducing the bulk acidity (Fig. 1a). However, this treatment also results in a more than fourfold increase in external acidity (Fig. 1d), which led to a reduction in T_{max} by more than 40 °C at low catalyst loading (Fig. 1b). Apparently, a treatment that reduces gas cracking activity can noticeably increase plastic cracking activity. This shows that external acidity can serve as a design criterion in the development of new polyolefins cracking catalysts. (...) Importantly, the external acidity fails to capture the remaining activity trends. ZY₅₅ and ZY₁₄ showed similar external acidities, yet their T_{max} at low catalyst loading deviated by >20 °C (Fig. 1b). Similarly, the non-steamed ZY₅ shows comparable external acidity but noticeably lower activity compared with the other zeolites. However, here the decreased activity might be explained in a similar fashion as its reduced gas cracking activity (Fig. 1a), namely by the lower amount of mesoporosity of the non-steamed ZY₅ compared to the other zeolites and the higher acid strength of steamed zeolites. (...) To study the remaining differences in activities, we opted to conduct more in-depth kinetic experiments.”*

In page 3, the authors state that the external acidity of the catalyst is more favorable for polyolefin conversion, can it be further demonstrated experimentally that the internal acid sites of the zeolite have less of an effect?

Completely isolating the effect of active sites located inside or outside of micropores for a zeolite catalyst is very difficult. One approach would be to selectively poison either of these sites. We attempted to selectively poison the external sites by adsorption of TTBP on ZY₃₆. As we were unable to detect a noticeable drop in activity, we reason that this strategy is not adequately poisoning the external sites. Due to the hydrophobic tert-butyl groups, TTBP might dissolve readily in the plastic melt at the temperatures required for cracking, leaving the acid site and making it available for cracking. For

proper poisoning, full removal of external acid site protons, e.g., by exchange with sodium, would be required. We believe that development of such a procedure with convincing isolation of external/internal acid sites would present a challenging work in its own right, and as such is beyond the scope of the study.

The less important role of internal sites is furthermore also evident from the selectivity data. The bulk BAS for ZY₁₂ is more than three times larger than for ZY₅₅, yet the selectivity for both materials is remarkably similar, with the exemption of coking, suggesting the role of internal sites mainly affect coking and secondary cracking of vapors.

Are the TGA calculation predictions subject to large errors because of the large differences between the TGA ramp experiments and the semi-intermittent reactor experimental processes (including warming rates, reaction conditions, etc.)?

TGA and semi-batch reactor are good qualitative agreement for this study: The catalyst show remarkably similar activity in the semi-batch reactor (Fig. 4a), and the similar differences in coking as observed using TGA (Fig. 4c, Fig. S10). In general, both experiments show that coking increases with increasing BAS content, although ZY₅₅ shows higher coking in TGA experiments compared with ZY₅₆, while the opposite is true or the semi-batch experiments. When absolute coking levels are compared between TGA and semi batch reactor, the TGA experiments show lower coke yields, likely as a result of the shorter contact time of the hydrocarbon vapors with the zeolite. We therefore are of the opinion that the qualitative activity trends observed using TGA in this study are transferable to larger reactors operating with a temperature ramp on the gram scale, while quantitative coke yields are underestimated.

Fig. 3e, SEM cannot clearly show that the extended polymer chains can cover a large portion of the zeolite particles, please provide additional characterization to prove.

In this illustration, together with the calculation in supplementary note 4, we try to show that it is not unreasonable to assume that a polymer chain can interact with multiple acid sites. The figure provides a visual comparison between the size of the zeolite particles and schematically drawn extended polymer chains. We believe that for the stated purpose this illustration is sufficient.

We have adjusted the wording on page 7 to better reflect this.

Page 7:

On the outermost surface, extended polymer chains ~~can~~ could stretch over a large part of the zeolite particle. Fig. 3e shows an electron micrograph of ZY₁₄ particles, overlaid bars and dots showcasing the approximate length of an extended or coiled up polymer chains to illustrate the relative sizes of both. ~~As can be seen from a comparison of an extended chain and electron micrographs. We expect therefore that a~~ single polymer macromolecule can ~~therefore be expected to~~ interact with multiple acid sites simultaneously.

Furthermore, we expect that producing such a figure experimentally, i.e. depositing isolated polymer chains on a zeolite to be a highly difficult task, as the polymer begins the crack at the temperatures required to dissolve it in appropriate solvents like trichlorobenzene.

In Fig. 4b, explain why C4 and C5 have the highest selectivity in the gas?

The selectivity towards C4 and C5 hydrocarbons is largest in the gas phase, as higher hydrocarbons are caught in the cold traps at 0 °C. For the shorter hydrocarbons, i.e. C1-C3, only propylene is likely

produced by chain-end scission¹, while the other light gases are produced in secondary reactions from larger hydrocarbons, which are actively removed from the reactor. We expect that with lower carrier gas flows, and therefore longer residence times, the selectivity towards these gases will increase.

The following additional changes were made to the main manuscript and the SI:

- The Acknowledgments were amended:
B.M.W. and I.V. are supported by ~~as well as from~~ the Advanced Research Center (ARC) Chemical Buildings Blocks Consortium (CBBC)
- An additional affiliation was added for Z.M.R.
²ENS de Lyon, Département de Chimie, 46 allée d'Italie, 69007 Lyon, France
- In the methods section, the location of the N-H stretch vibration was added
(...) the N-H stretch vibration located at ~~XX~~ 3369 cm⁻¹ was quantified (...)
- Figure S13 was renamed to Fig. S14.
- The data availability section has been split into data and code availability sections.

References:

1. Rejman, S. *et al.* Transport limitations in polyolefin cracking at the single catalyst particle level. *Chem. Sci.* **14**, 10068–10080 (2023).
2. Hayhurst, D. T. & Paravar, A. R. Diffusion of C1 to C5 normal paraffins in silicalite. *Zeolites* **8**, 27–29 (1988).
3. Hor, J. L., Wang, H., Fakhraai, Z. & Lee, D. Effect of Physical Nanoconfinement on the Viscosity of Unentangled Polymers during Capillary Rise Infiltration. *Macromolecules* **51**, 5069–5078 (2018).
4. Martens, J. A. *et al.* Evidences for pore mouth and key–lock catalysis in hydroisomerization of long n-alkanes over 10-ring tubular pore bifunctional zeolites. *Catal. Today* **65**, 111–116 (2001).
5. Macko, T., Pasch, H. & Brüll, R. Selective removal of polyethylene or polypropylene from their blends based on difference in their adsorption behaviour. *J. Chromatogr. A* **1115**, 81–87 (2006).
6. Berger, F., Rybicki, M. & Sauer, J. Adsorption and cracking of propane by zeolites of different pore size. *J. Catal.* **395**, 117–128 (2021).