# Supplementary material for: External acidity as performance descriptor in polyolefin cracking using zeolite-based materials
Source: Nat Commun. 2025 Mar 26;16:2980. doi: 10.1038/s41467-025-57158-1 (PMC11947190; doi:10.1038/s41467-025-57158-1)
Supplement: Supplementary file 1 — Supplementary Information [file 41467_2025_57158_MOESM1_ESM.pdf]

Supplementary Information: External Acidity as Performance Descriptor in Polyolefin Cracking using Zeolite-Based Materials

Sebastian Rejman[1], Zoe M. Reverdy[1,2], Zeynep Bör, [1] Carolin Rieg,[1] Joren M. Dorresteijn,[1] Jan-Kees van der Waal[3], Eelco T.C. Vogt[1], Ina Vollmer[1,*], Bert M. Weckhuysen[1,*]

[1]Inorganic Chemistry and Catalysis group, Institute for Sustainable and Circular Chemistry, Department of Chemistry, Utrecht University, Universiteitsweg 99, 3584 CG Utrecht, The Netherlands.

[2]ENS de Lyon, Département de Chimie, 46 allée d'Italie, 69007 Lyon, France[3]TNO, Pieter Calandweg 15, 2628 CP Delft, The Netherlands

Contents

Supplementary notes 2

Supplementary note 1: Density Functional Theory (DFT) calculations 2

Supplementary note 2: Deviations from 1st order kinetics 3

Supplementary note 3: Discussion of deviations between Hg porosimetry and Ar physisorption 3

Supplementary note 4: Estimation of average acid site proximity 3

Supplementary note 5: Discussion of coking effects 3

Supplementary Figures 4

Pyridine-IR spectroscopy 4

Thermogravimetric analysis weight loss profiles 5

Supplementary References 17

Supplementary notes

Supplementary note 1: Density Functional Theory (DFT) calculations

To gain insights on the differences between cracking in the micropores of a Y zeolite and on the zeolite external surface, we computed the activation and reaction energy of the first step in the cracking of an n-hexane molecule: the insertion of a proton into the middle C-C bond, creating a non-classical carbonium ion, the first step in the cracking reaction from hexane to propane and propene. We considered several models (all given as .cif files attached to this PDF).

1. A primitive unit cell (48 T atoms) with a single acid site (47 Si and 1 Al).
2. A primitive unit cell with 36 Si and 12 Al atoms (and charge compensating protons); the distribution of Al over the lattice was determined previously to match with ^{27}Al NMR results.[1]
3. A primitive unit cell with 44 Si and 4 Al atoms; the Al atoms are all part of the same hexagonal prism, which was also present in model 2.
4. Model 3 with a La^{3+} ion in the hexagonal prism, replacing three protons.
5. A primitive unit cell with 41 Si and 7 Al atoms; this contains the Al substituted hexagonal prism of model 3 and a nearby hexagonal prism with three Al. The substructure with these two hexagonal prisms was taken from model 2.
6. As model 5, but with a La^{3+} ion in the second hexagonal prism, replacing all protons.
7. A slab representing the surface of the Y zeolite with a single Al substitution and a charge compensating proton.

For models 1-6 the unit cell lattice constant was optimized before introduction of hexane and kept fixed afterwards. The 2D lattice of the slab model was based on the unit cell dimensions of all-silica faujasite.

In all cases, we started from an n-hexane molecule sorbed on a proton on the O, connected to Al, that bridges the two rings of the hexagonal prism. To allow a proper comparison, in models 2-6 the same site on the hexagonal prism with 4 Al was targeted.

Table S1: The activation and reactions energies in kJ/mol units.

model	Activation energy (kJ/mol)	Reaction energy (kJ/mol)
1	146.9	136.0
2	211.71[1]	211.5
3	187.9	181.2
4	158.2	143.1
5	190.0	184.5
6	188.7	178.7
7	170.7	155.6

The lowest activation and reaction energy are found for model 1, a primitive unit cell with a single acid site (SAR = **94**). From this it can be seen that the presence of nearby protons is detrimental to the reaction. In the unit cell, with twelve acid sites (model 2, SAR = 6), the activation energy (for the targeted site) has risen from 146.9 to 211.71 kJ/mol.

With only three more protons, but on the same hexagonal prism (model 3), the activation energy has risen from 146.9 to 187.9 kJ/mol. Introducing a La^{3+} ion (model 4) and thereby losing those three protons, lowers the activation energy to 158.2 kJ/mol. That the effect of introducing La is based on replacing nearby protons becomes clear by comparing models 5 and 6, where this has little influence. The additional protons in model 5 are relatively far from the targeted site and replacing these has only a minor influence on activation and reaction energies.

The activation and reaction energy on the outer surface model (7) are larger than for an isolated acid site, but smaller than within a micropore of a zeolite with low SAR. Whereas in a micropore confinement is favorable to cracking, the presence of nearby protons destabilizes the carbonium ion intermediate which increases both activation and reaction energies.

[1] An extra decimal is used to show the (very small) difference between activation and reaction energy.

Supplementary note 2: Deviations from 1st order kinetics

For all catalyst materials under study, the isothermal thermogravimetric analysis (TGA) weight-loss profiles deviated from ideal first order kinetics (Fig. 2a main manuscript, Fig.S3). This is most evident at conversions < 25%, where the cracking rate is noticeably higher. We hypothesized two explanations of this behavior:

1. The kinetics of plastic cracking can be considered analogously to the kinetics of free radical polymerization, with an initiation, propagation and termination step. First order kinetics are only expected when the rate of termination and initiation are equal, i.e. the steady state approximation assuming a stable concentration of active centers holds. Quick deactivation of the most active sites at the start of the reaction could lead to the observed initial drop in reaction rate. This rests on the assumption that more active sites also deactivate faster.
2. As discussed in the main manuscript, the location of the bonds cracked can have a critical influence on the reaction kinetics. If the location of cracked bonds along the polymer backbone changes over the course of the reaction, e.g. from mid-of-chain to end-of-chain, a change in rate will be observed in weight-loss kinetics.

To test both hypotheses, an experimental approach could involve extraction of residual polymer from the zeolite catalyst at different stages of the reaction. By studying coke deposits and cracking activity of the extracted catalyst, deactivation over the course of the reaction could be investigated. By studying the molecular weight distribution of the extracted polymer, it might be possible to gain insight over the location of cracked bonds. More sophisticated kinetic models using a termination and initiation step might also help explain the deviations. These studies are beyond the scope of this work.

Supplementary note 3: Discussion of deviations between Hg porosimetry and Ar physisorption

Since both methods rely on fundamentally different principles, deviations are expected, however the discrepancy could also be caused by a majority of the mesopore volume being constricted. In the pressure regime utilized, only pores up to 3.6 nm in diameter can be probed, meaning that if a large share of pore volume is only accessible through pores narrower than 3.6 nm, Hg cannot enter and less mesopore volume compared to the physisorption will be observed. This type of constriction can be studied using Ar physisorption at temperatures below 77 K and hysteresis loop cycling,[2,3] however this is beyond the scope of this study. This type of constriction could prove significant to plastic conversion, as it could significantly limit mass transport and necessitate characterization more in depth-mesopore characterization.

Supplementary note 4: Estimation of average acid site proximity

To estimate the average distance between external acid sites, we assume them to be distributed evenly in a hexagonal pattern. The surface area of a hexagon A is described by $A = \sqrt{3/4} \cdot a$ where a is the side length. The inradius r is described by $r = \sqrt{3/2} \cdot a$. With the average distance between hexagon centers $d = 2r$, this yields Eq. S1:

$$d = 2 \cdot \sqrt[4]{3} \cdot \sqrt{A} \qquad (Eq.S1)$$

The average area for each external acid site is calculated as $A = SA_{\text{ext}} / n_{BAS,ext}$ where SA_{external} is the area determined by NL-DFT for pores smaller than 2 nm and $n_{BAS,ext}$ is determined by TTBP-IR. Therefore, the average distance between external acid sites can be estimated as:

$$d = 2 \cdot \sqrt[4]{3} \cdot \sqrt{\frac{SA_{\text{ext}}}{n_{\text{BAS,ext}}}} \qquad (Eq.S2)$$

Using the ZY_{14} material with $SA_{\text{ext}} = 74\ m^2/g$ and $n_{BAS,ext} = 35$ µmol/g yields an approximate distance of 5 nm.

Supplementary note 5: Discussion of coking effects

In non-isothermal thermogravimetric analysis (TGA) experiments, the coke yield (m(coke)/m(polymer)) was determined by burning off the catalyst material after each cracking experiment (Fig. S10). Coke yield increased with catalyst loading for all catalyst materials under study, ranging from 0.3% for ZY_{56} at lowest catalyst loading, to 10% for ZY_{5B} at highest loading. In general, a catalyst material with a higher aluminum content shows a higher coke yield. This can be explained by coke formation being a secondary reaction of shorter hydrocarbons that can enter into the catalyst micropores more easily (see main manuscript). An exemption to this trend is ZY_{55}, which shows a higher coke yield compared to ZY_{47} and ZY_{56}, although the difference is small.

Supplementary Figures

Pyridine-IR spectroscopy

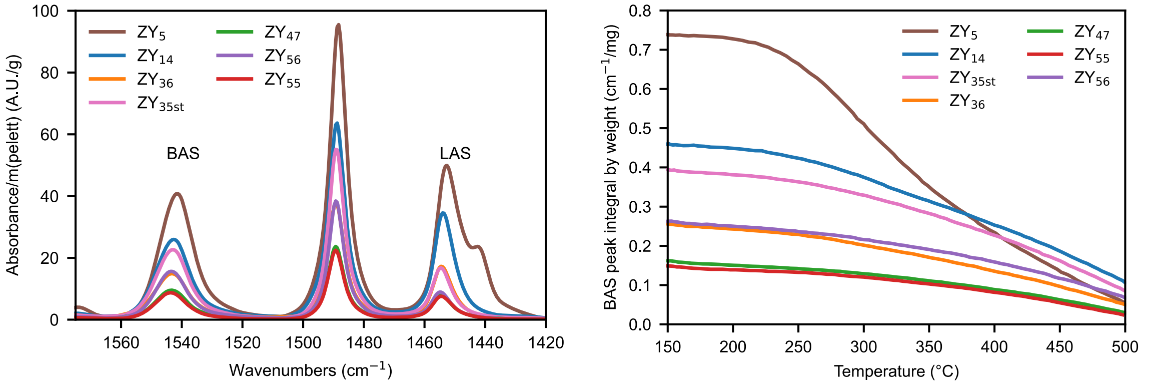

Fig. S1: Left: Infrared (IR) spectra of pyridine adsorbed on the different zeolite materials under study (ZY_5-ZY_{56}) normalized by pellet weight showing the characteristic vibrations of protonated pyridine (BAS) and pyridine coordinated to Lewis acid sites (LAS). Right: Temperature programmed desorption (TPD) of pyridine showing the BAS peak area normalized by pellet weight as a function of the measurement temperature. The number of acid sites in not quantified as the extinction coefficient is temperature dependent.[4]

Thermogravimetric analysis weight loss profiles

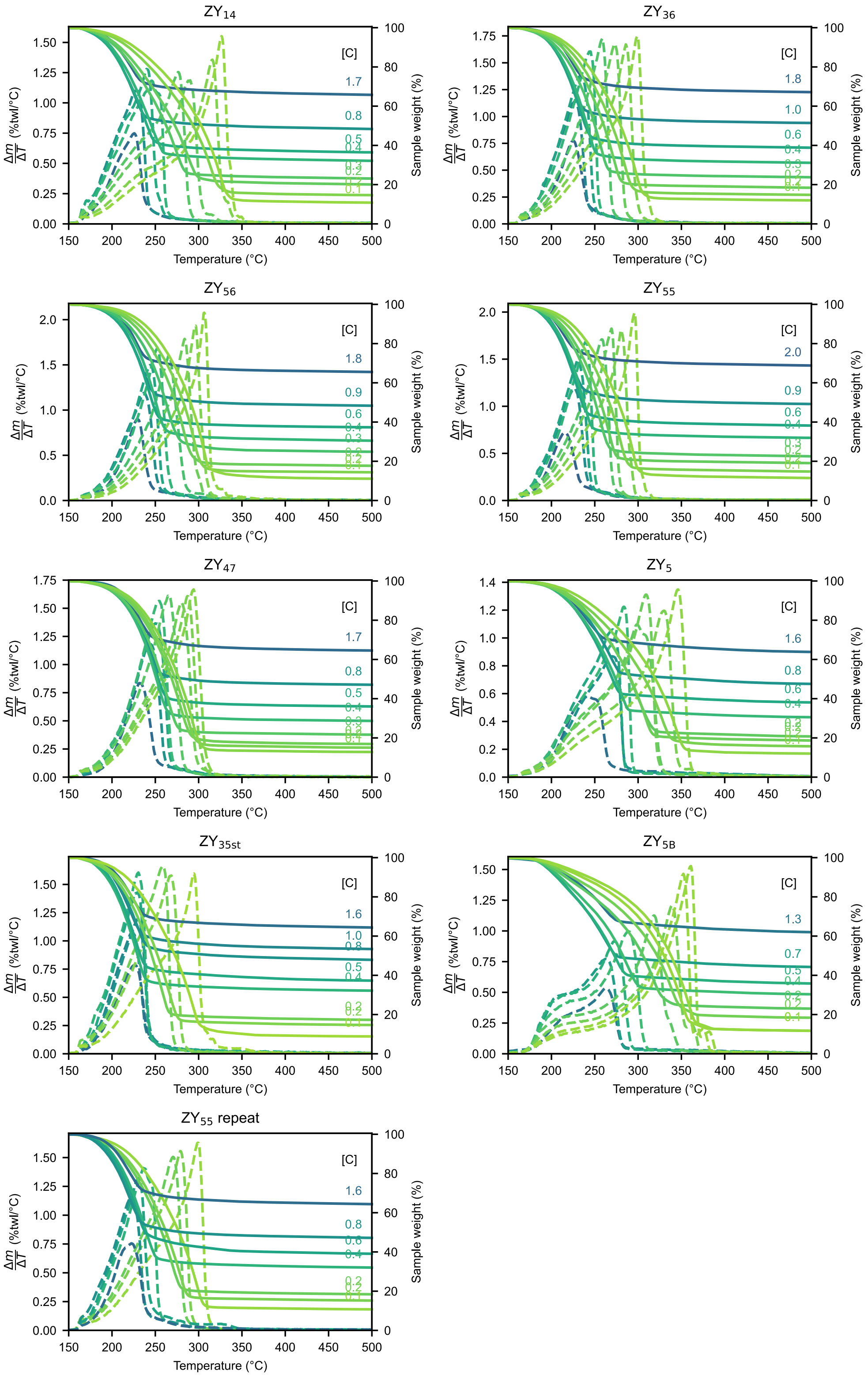

Fig. S2: Non-isothermal thermogravimetic analysis (TGA) profiles for the cracking of polypropylene (PP) using the different zeolite materials under study (ZY_5-ZY_{56}). twl: total weight loss. [C]: m(Catalyst)/m(Polymer).

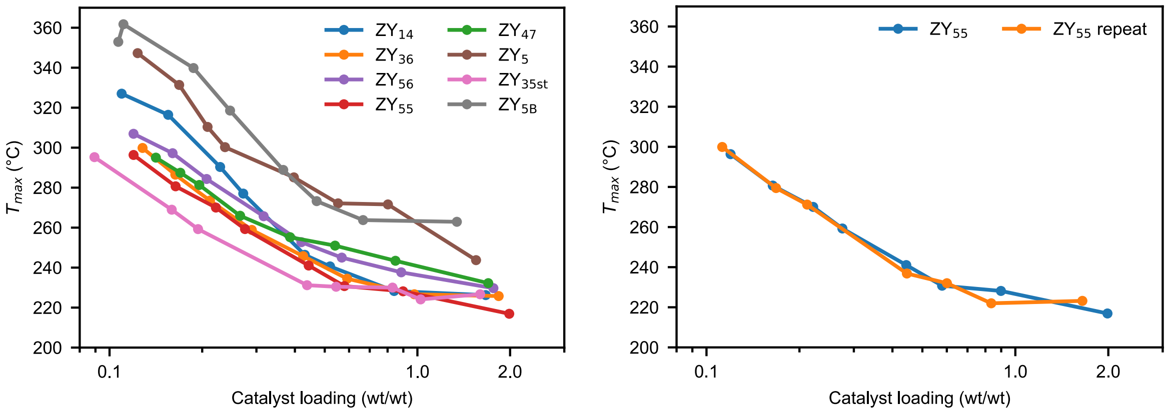

Fig. S3: Temperarature of highest cracking rate T_{max} in cracking of polypropylene (PP) determined by ramped thermogravimetric analysis (TGA) of polymer-catalyst mixtures at different catalyst loadings and the different zeolite materials under study (ZY_5-ZY_{56}). Lower T_{max} indicates higher cracking activity. See Fig. S2 for full TGA profiles. Catalyst ZY_{5B} was obtained by transfering a Zeolyst CBV300 zeolite to proton form by calcination for 24 h at 550 °C. The ZY_{55} repeat was conducted

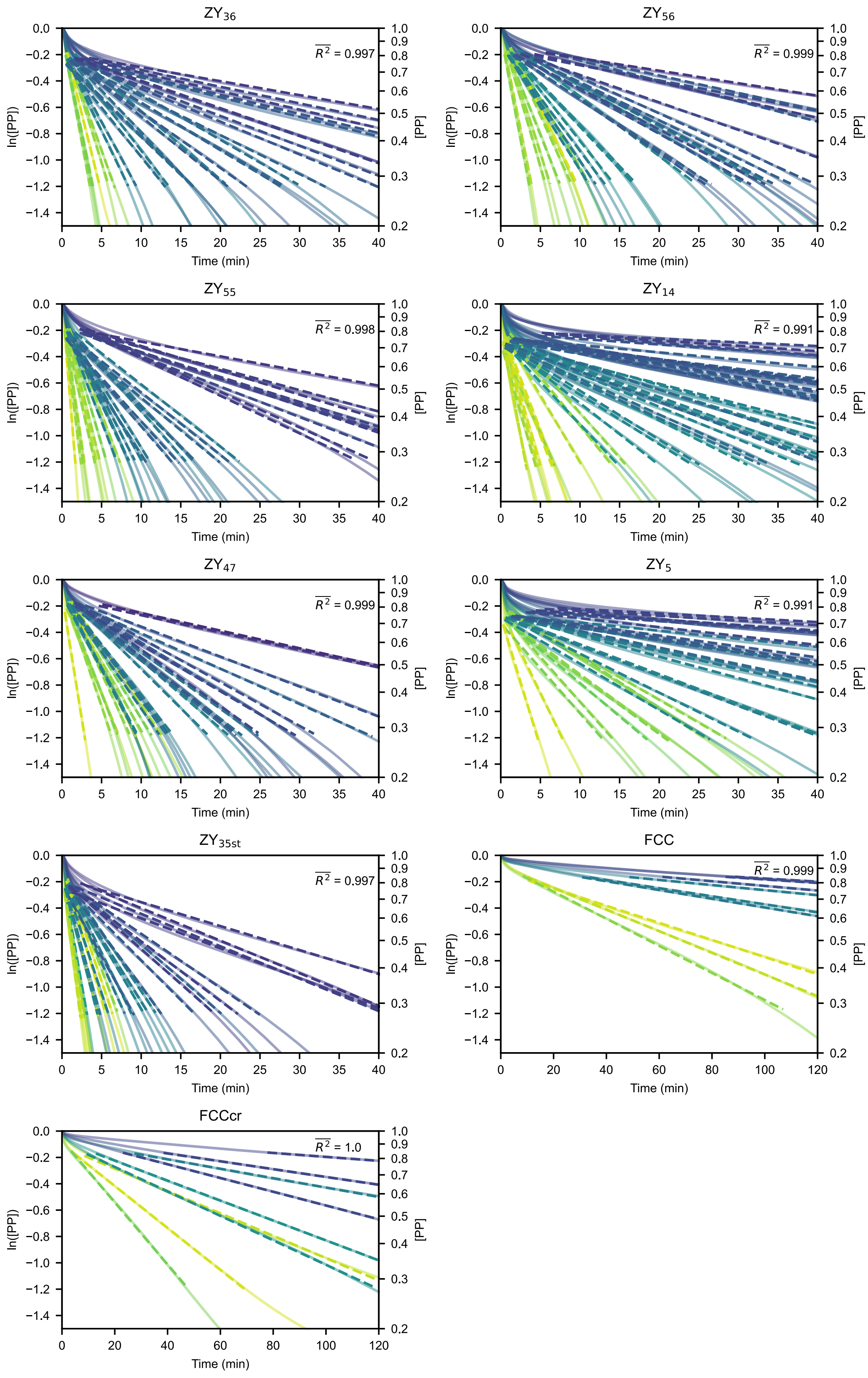

Fig. S4: Isothermal thermogravimetric analysis (TGA) profiles for cracking of polypropylene (PP) at different temperatures and catalyst loadings for all zeolite materials under study (ZY_5-ZY_{56}) and a comparison with the fluid catalytic cracking

(FCC) materials. Color change from purple to yellow indicates increasing temperature. R^2 shown is averaged over all experiments.

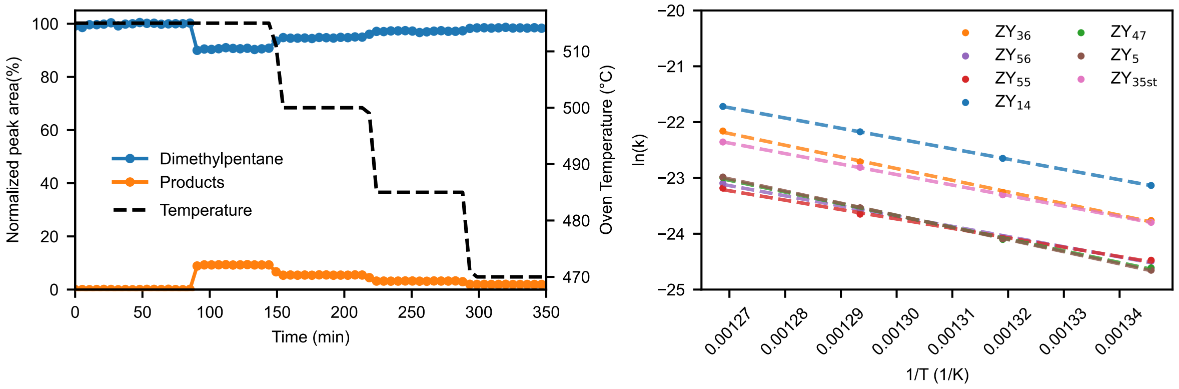

Fig. S5: Left: Temperature, dimethylpentane (DMP) and product concentrations for DMP cracking using the ZY_{47} material. Right: Arrhenius plots for DMP cracking using the different zeolite materials under study (ZY_5-ZY_{56}).

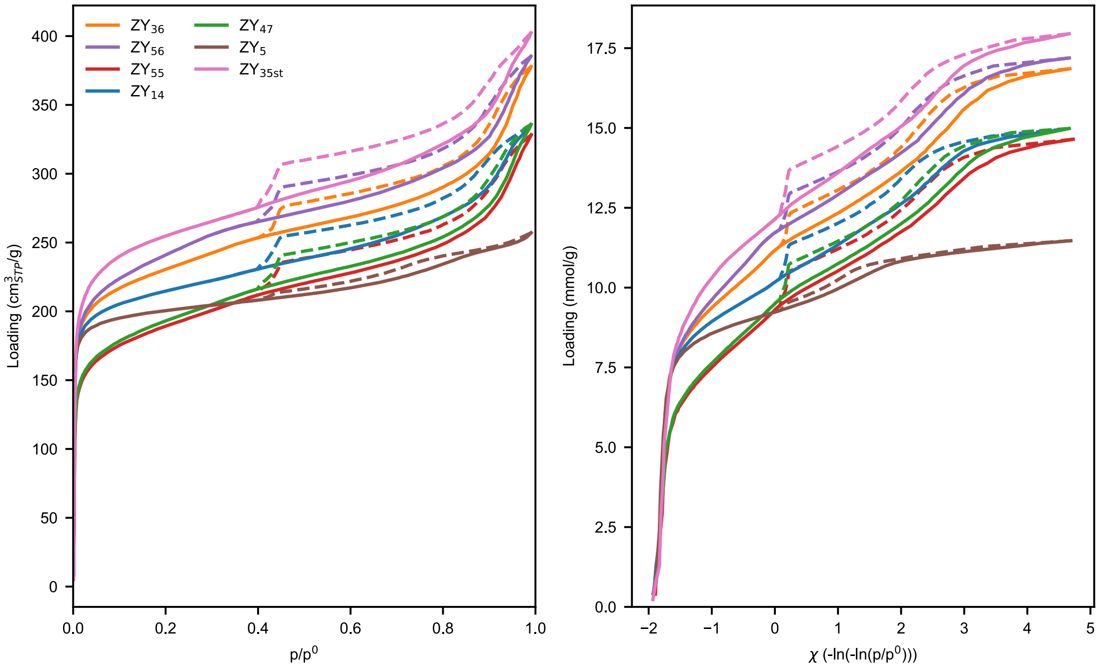

Fig. S5: Ar isotherms at 87 K plotted as a function of p/p^0 and as χ plot ($-\ln(-\ln(p/p^0)$) for the different zeolite materials under study (ZY_5-ZY_{56})

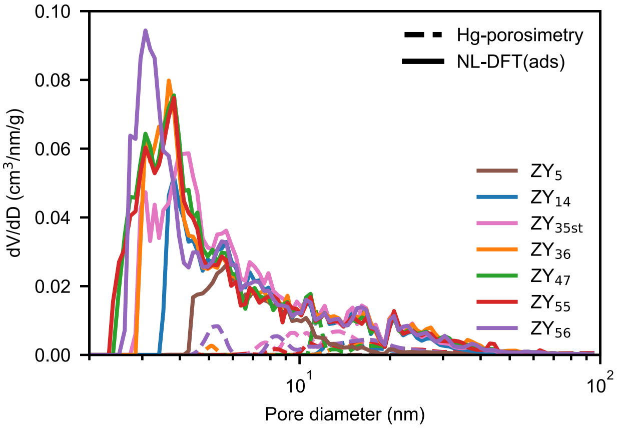

Fig. S6: Pore size distribution for all studied zeolite materials (ZY_5-ZY_{56}) determined by non-local density functional theory (NL-DFT) of the adsorption branch of Ar physisorption at 87 K using a hybrid kernel of spherical micropores and cylindrical mesopores as well as Hg porosimetry.

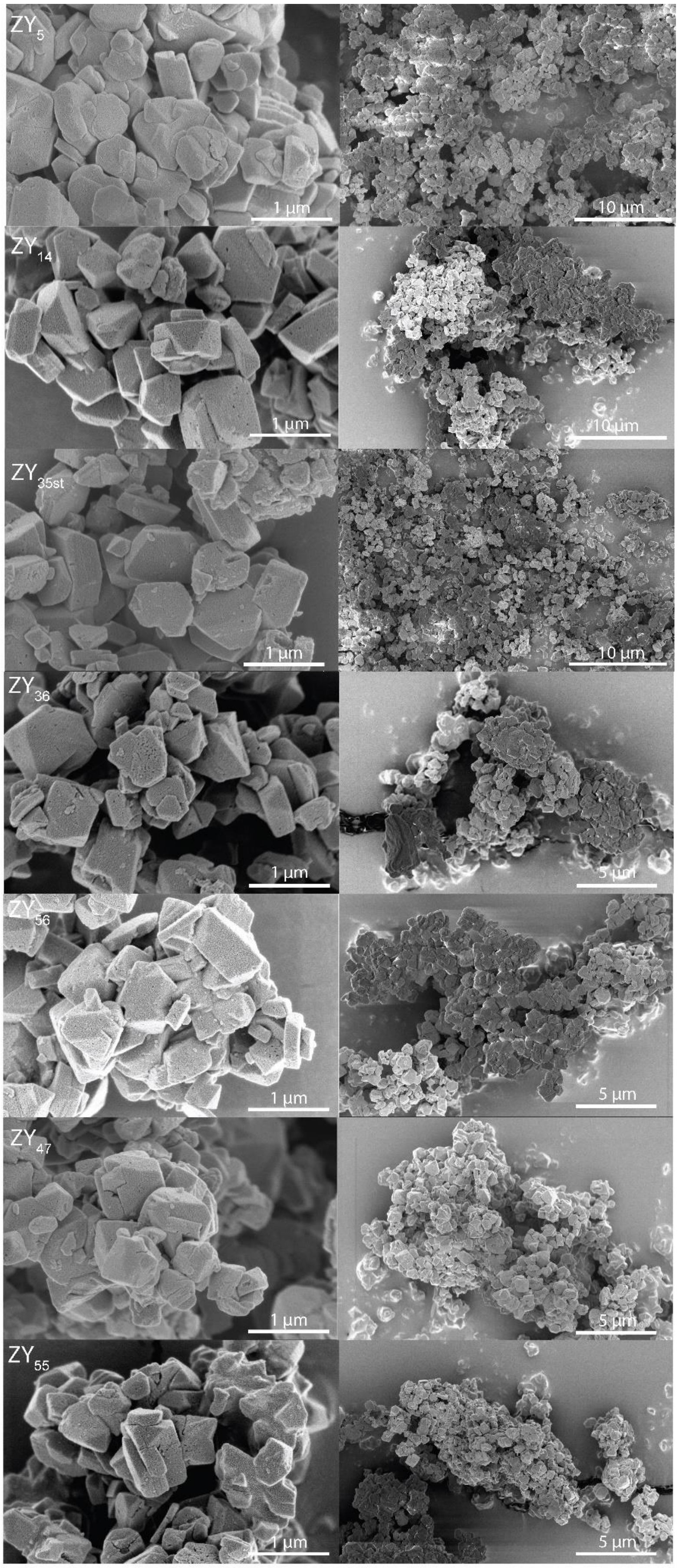

Fig. S7: Scanning electron micrographs (SEM) of all zeolite materials (ZY_5-ZY_{56}) studied in this work.

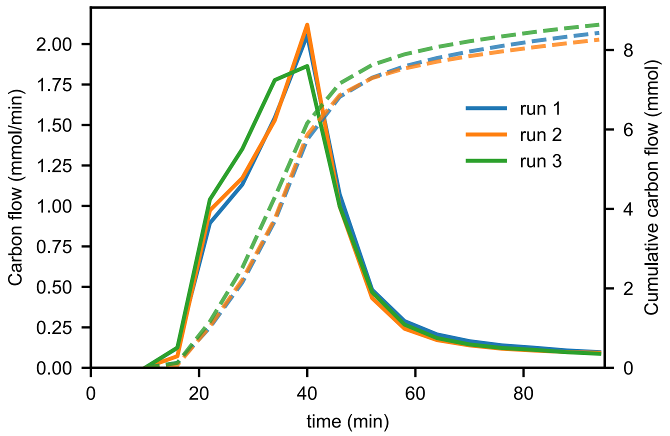

Fig. S8: Evolution of non-condensed hydrocarbons for cracking of polypropylene (PP) using the ZY_{56} material in a semi-batch reactor determined by on-line gas chromatography (GC). Three experiments were conducted under identical conditions.

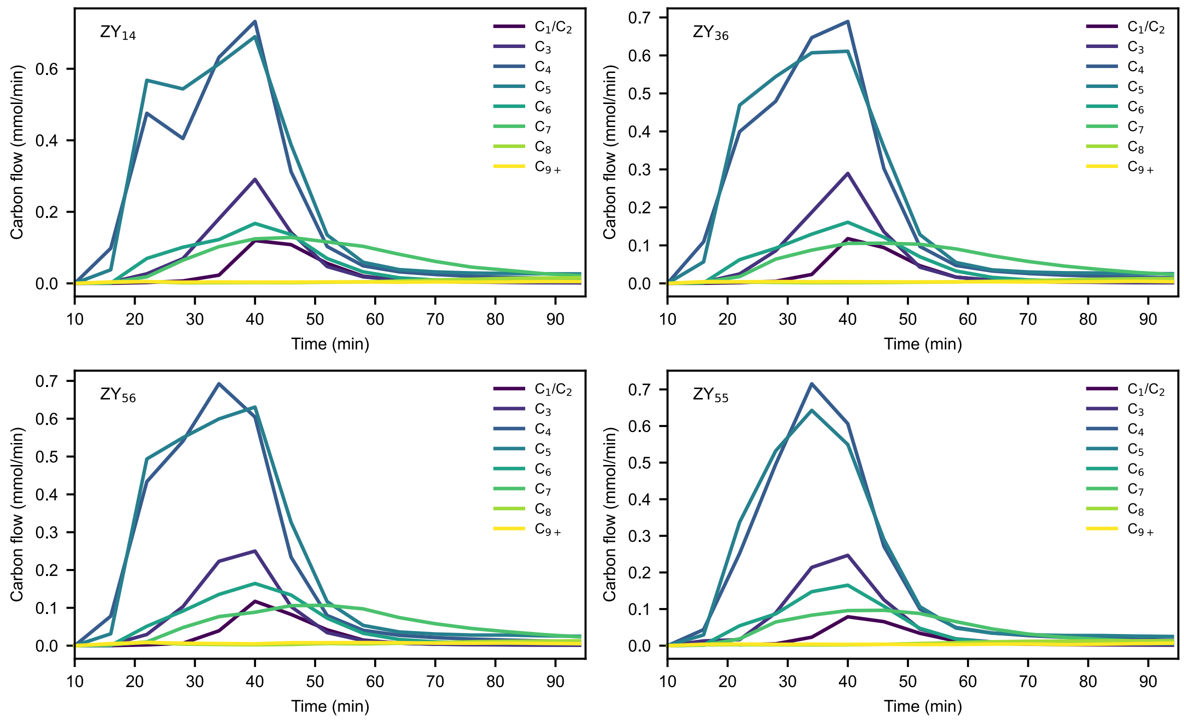

Fig. S9: Evolution of non-condensed hydrocarbons for cracking of polypropylene (PP) using different zeolite materials under study (ZY_{14}-ZY_{56}) in a semi-batch reactor determined by on-line gas chromatography (GC).

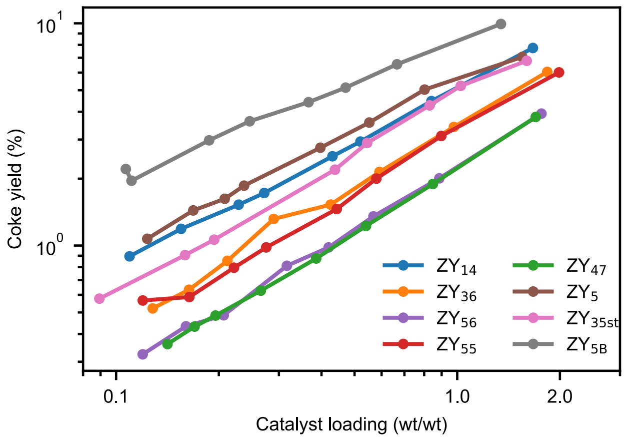

Fig. S10: Coke yield determined by non-isothermal thermogravimetric analysis (TGA) of polymer:catalyst mixtures for the different zeolite materials under study (ZY_5-ZY_{56}).

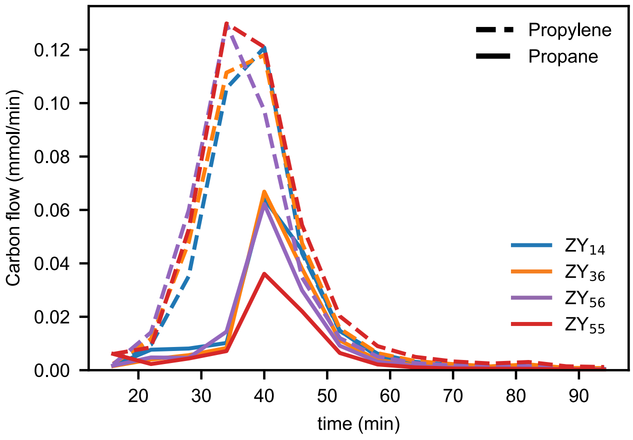

Fig. S11: Propane/propene flows in cracking of polypropylene (PP) using the different zeolite materials under study (ZY_{14}-ZY_{55}).

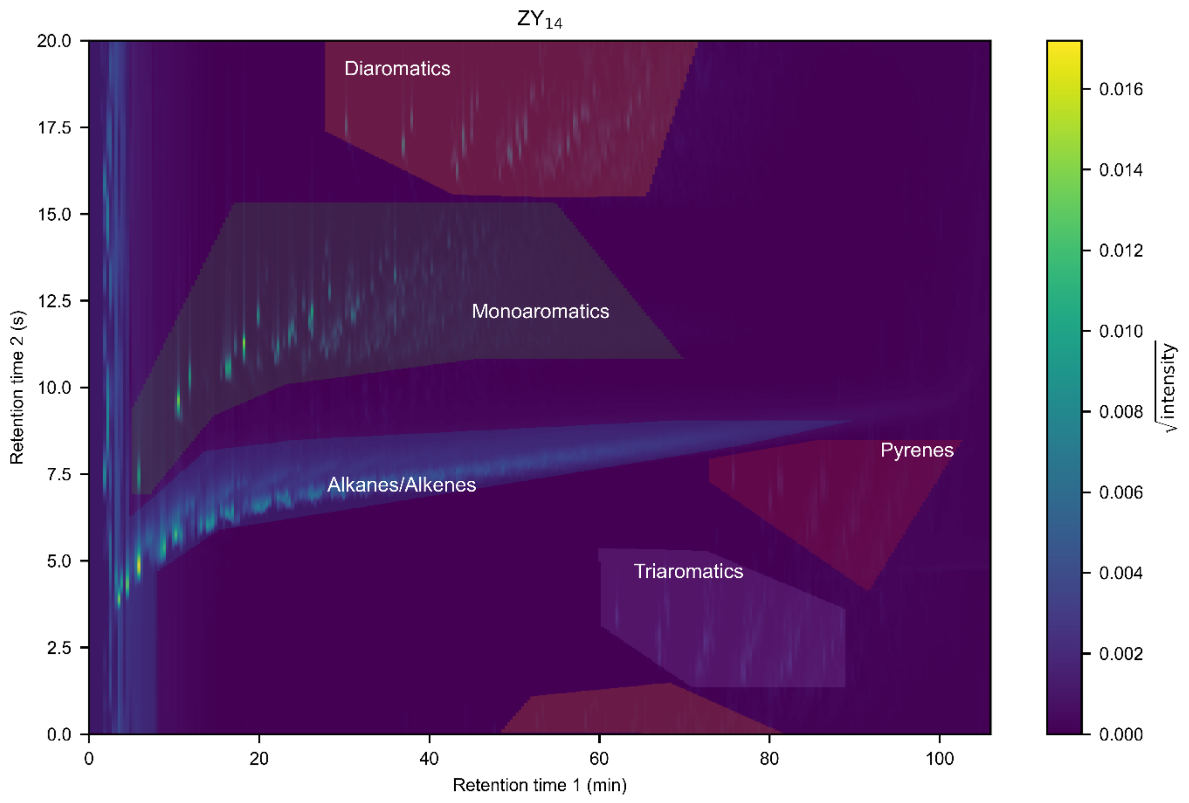
ZY14
Diaromatics
Monoaromatics
Alkanes/Alkenes
Pyrenes
Triaromatics
Retention time 2 (s)
Retention time 1 (min)
√intensity

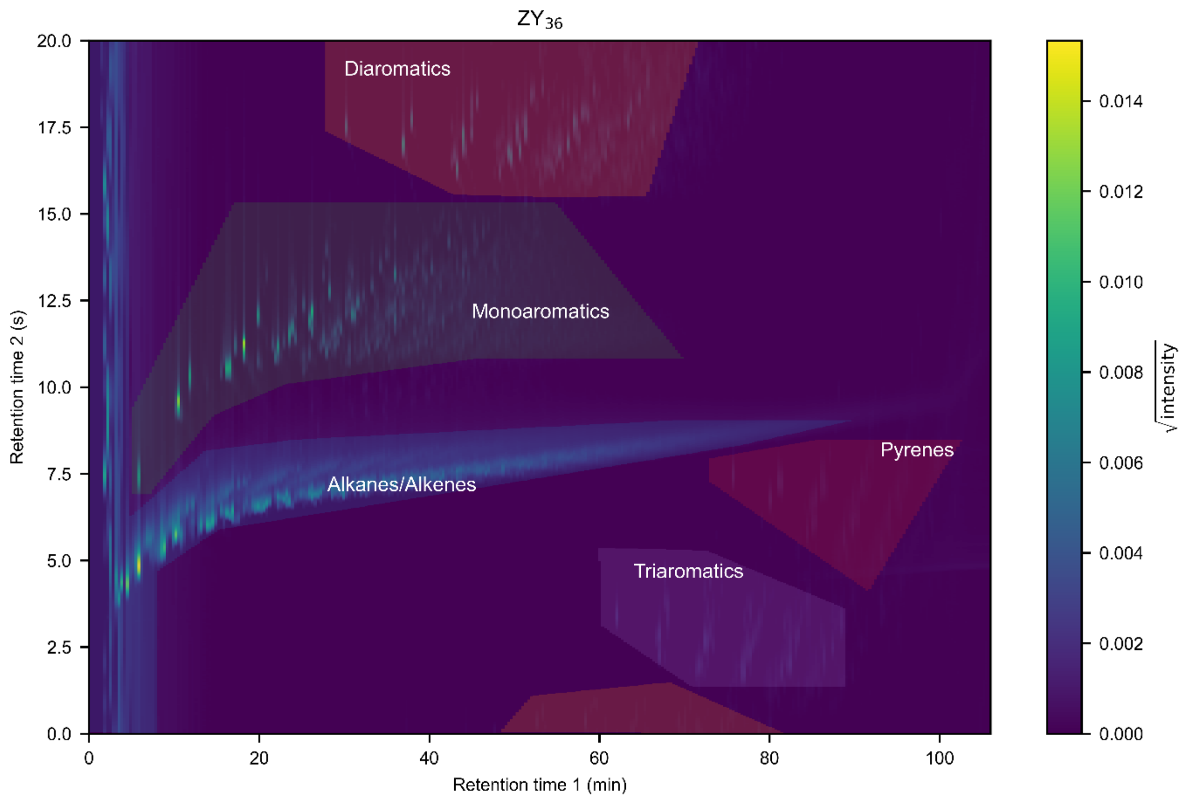
ZY36
Diaromatics
Monoaromatics
Alkanes/Alkenes
Pyrenes
Triaromatics
Retention time 2 (s)
Retention time 1 (min)
√intensity

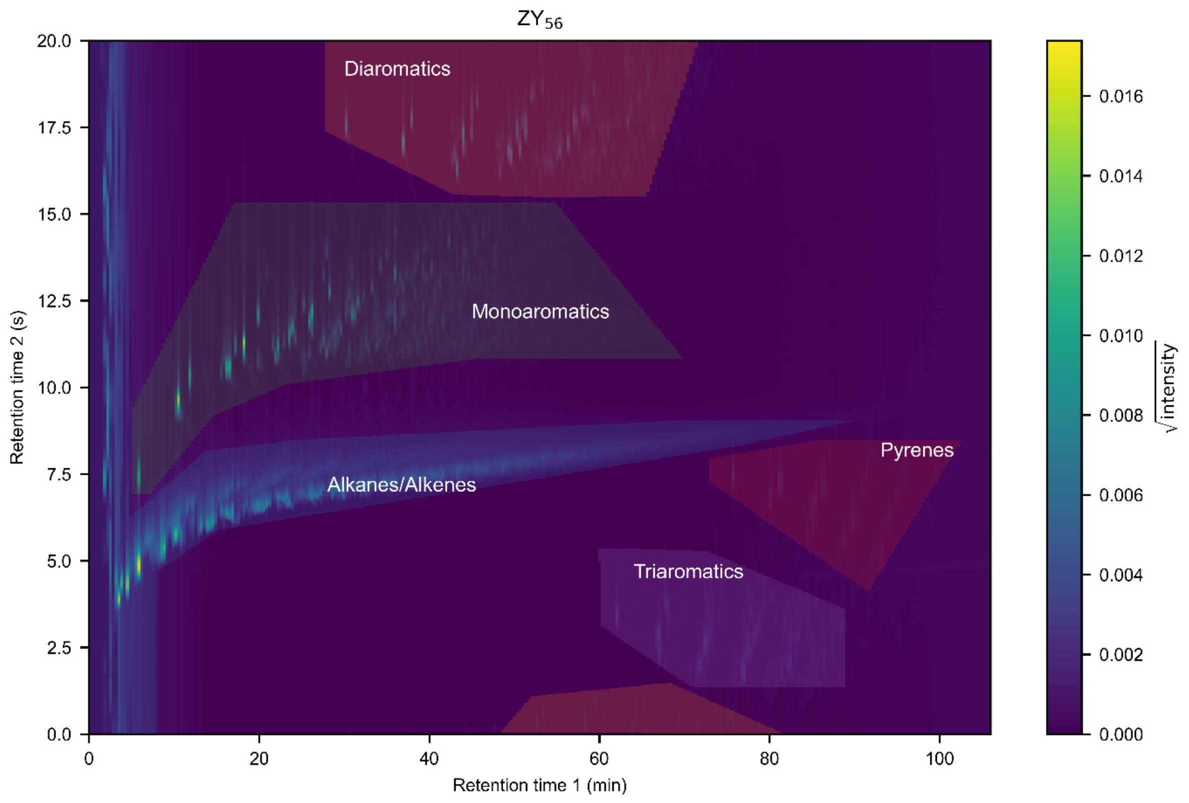

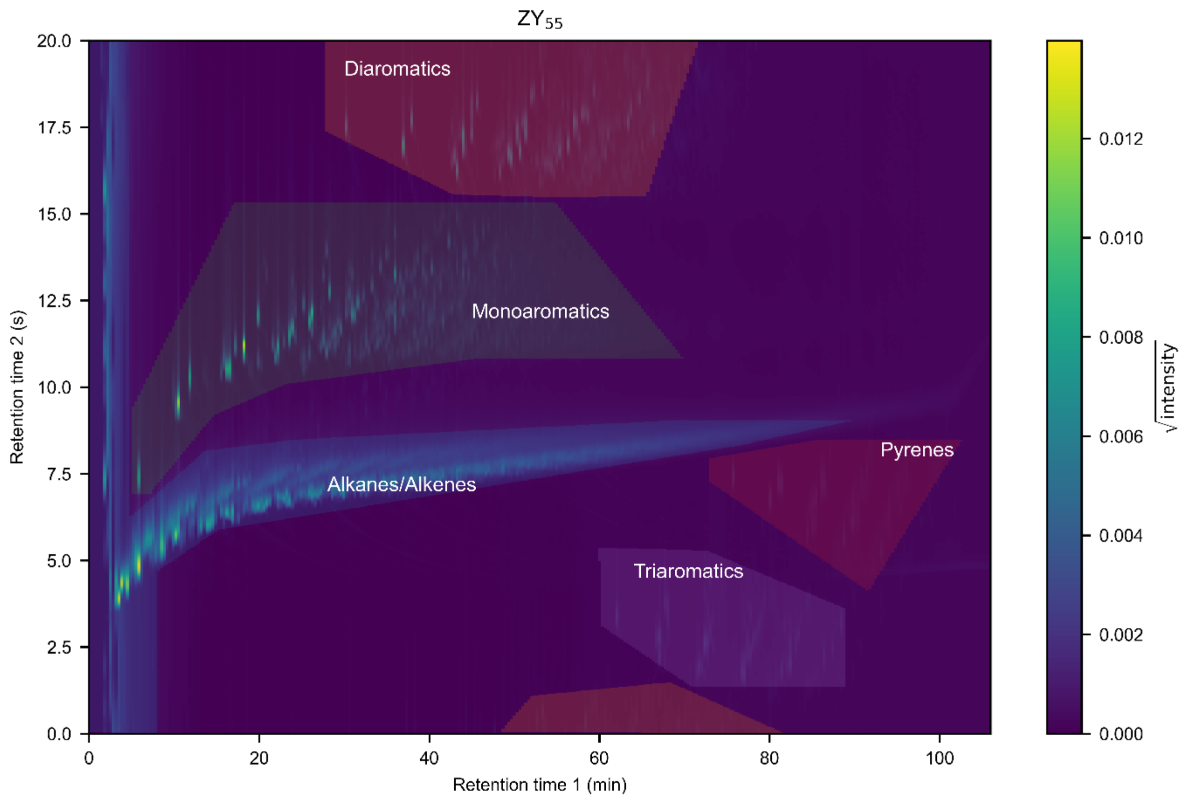

Fig. S12: 2D gas chromatograms (GC) of pyrolysis oil obtained by cracking of polypropylene (PP) using the different zeolite materials under study (ZY_{14}-ZY_{56}).

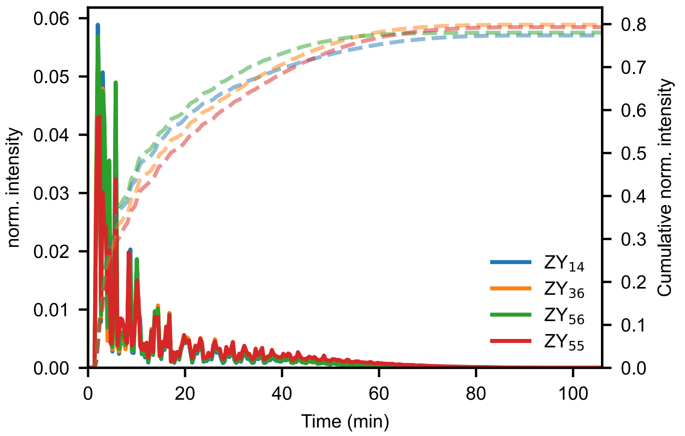

Fig. S13: Comparison of boiling point distribution of alkanes/alkenes in the pyrolysis oils by integration of masked 2D chromatograms.

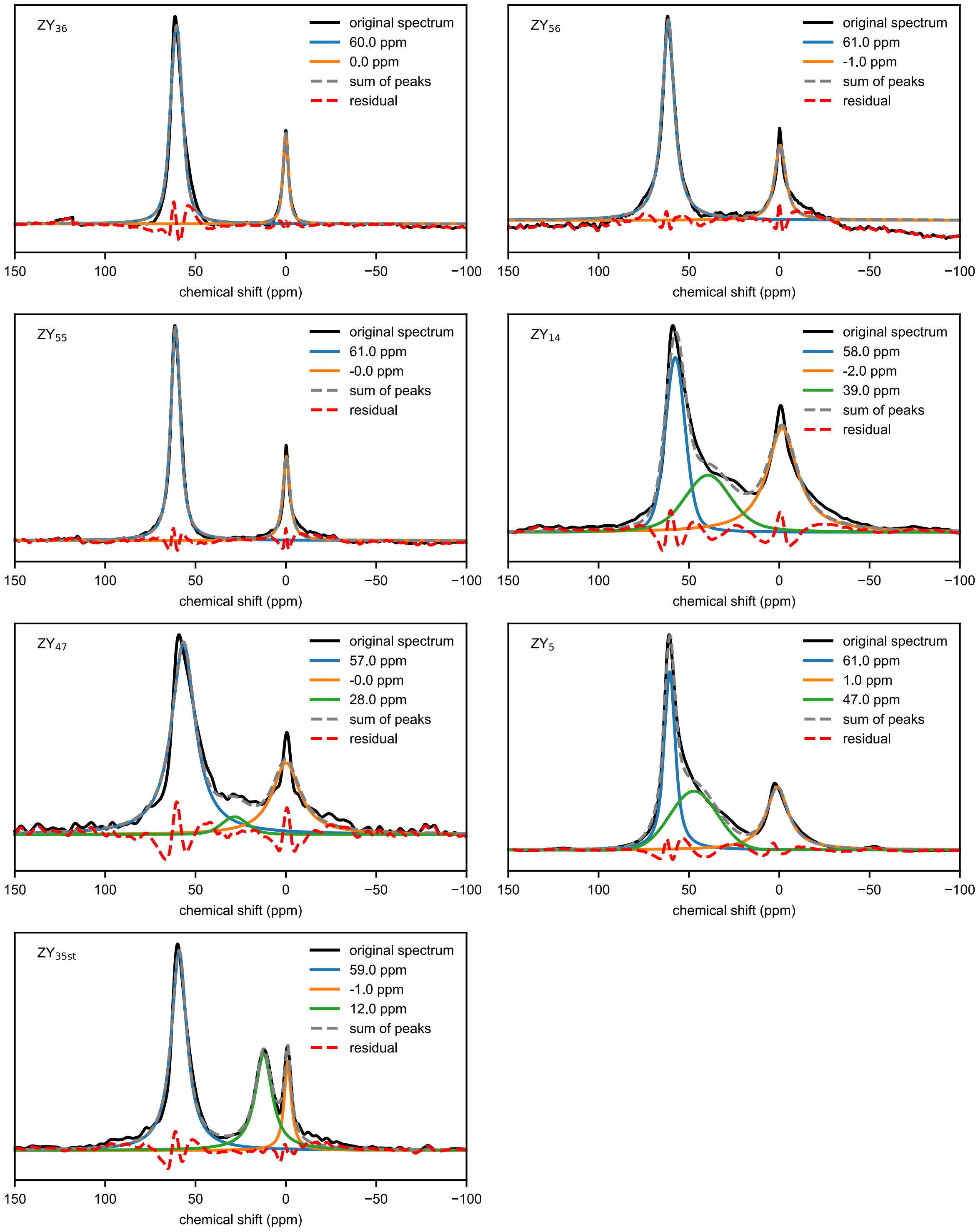

Fig. S14: ^{27}Al-magic angle spinning (MAS) solid-state nuclear magnetic resonance (NMR) spectra for all zeolite materials under study (ZY$_5$-ZY$_{56}$), including the fitted peaks, the sum of the peaks and the residual spectrum.

Supplementary References

1. Louwen, J. N. *et al.* Role of Rare Earth Ions in the Prevention of Dealumination of Zeolite Y for Fluid Cracking Catalysts. *J. Phys. Chem. C* **124**, 4626–4636 (2020).
2. Kenvin, J. *et al.* Quantifying the Complex Pore Architecture of Hierarchical Faujasite Zeolites and the Impact on Diffusion. *Adv. Funct. Mater.* **26**, 5621–5630 (2016).
3. Garcia-Martinez, J. *et al.* Evidence of intracrystalline mesostructured porosity in zeolites by advanced gas sorption, electron tomography and rotation electron diffraction. *ChemCatChem* **6**, 3110–3115 (2014).
4. Zholobenko, V. *et al.* Probing the acid sites of zeolites with pyridine: Quantitative AGIR measurements of the molar absorption coefficients. *J. Catal.* **385**, 52–60 (2020).
